# Association between IL-4 and IL-4R Polymorphisms and Periodontitis: A Meta-Analysis

**DOI:** 10.1155/2017/8021279

**Published:** 2017-03-14

**Authors:** Xiao-Wei Jia, Ya-Di Yuan, Zhong-Xiong Yao, Chang-Jing Wu, Xiang Chen, Xue-Hong Chen, Ying-Mei Lin, Xiang-Yu Meng, Xian-Tao Zeng, Jun Shao

**Affiliations:** ^1^Department of Stomatology, Guangzhou Hospital of Integrated Traditional and West Medicine, Guangzhou 510800, China; ^2^Center for Evidence-Based and Translational Medicine, Zhongnan Hospital of Wuhan University, Wuhan 430071, China

## Abstract

*Background*. Previous studies have revealed that gene polymorphisms of inflammatory factors may influence the development or progression of periodontitis, a main cause of tooth loss in adults; however, due to limitations of individual studies, inconsistent findings were reported.* Objective*. To meta-analytically investigate the relationship between periodontitis and the Interleukin-4 (IL-4) and Interleukin-4 receptor (IL-4R) gene polymorphisms.* Methods*. Databases were searched for relevant case-control studies. After study selection based on the predefined selection criteria, methodological quality assessment and data extraction were conducted independently by two reviewers, before subsequent statistical analyses.* Results*. 37 studies involving 4,385 patients and 5,168 controls were included. All the studied IL-4 polymorphisms were not significantly associated with periodontitis, except the -33C/T (CT versus CC: OR = 0.50, 95% CI = 0.28–0.88) associated with reduced AgP susceptibility. Positive association was found between IL-4R Q551 polymorphism and periodontitis susceptibility in three genetic models (R versus Q: OR = 1.59, 95% CI = 1.14–2.22; QR versus QQ: OR = 1.84, 95% CI = 1.21–2.80; RR + QR versus QQ: OR = 1.82, 95% CI = 1.22–2.72).* Conclusions*. A positive association exists between the IL-4R Q551R polymorphism and occurrence of CP. The IL-4 -33 CT genotype is negatively associated with the occurrence of AgP.

## 1. Introduction

Periodontitis is by definition a chronic disease involving periodontal supporting tissues, the prevalence of which in adults in the United States was estimated as 46% in 2009 to 2012 [1]. Periodontitis is one of the main causes of tooth loss in adults. The etiology of this disease is complicated, and various factors including microorganisms' invasion, host's health status, and external environmental factors are involved in its development [2]. Previous studies have revealed that gene polymorphisms of inflammatory factors may take part in the development and/or progression of periodontitis, by regulating relevant protein levels and activity [3]. Among these inflammatory factors, Interleukin-4 (IL-4), a cytokine involved in the process of inflammation, is closely associated with the pathogenesis of periodontitis through enhancing Th2 cell proliferation, suppressing Th1 cell proliferation, and downregulating Th1-mediated immune response [4]. IL-4, through binding to its specific receptor, that is, Interleukin-4 receptor (IL-4R), transmits signals into the cellular nucleus and exerts biological functions. IL-4R is a protein which consists of two heterogeneous subunits, that is, *α* chain and *γ*c chain. *α* chain transfers from IL-4 signals which was subsequently amplified by *γ*c chain [5].

Currently, studies investigating the association between IL-4 gene polymorphisms and periodontitis mainly focus on its promoter region. Among the polymorphisms studied, single nucleotide polymorphisms (SNP), including the -590C/T (rs2243250), -33C/T (rs2070874), -1099T/G (rs2243248), and the -70-bp variable number of tandem repeats (VNTR), are the most frequently reported [6, 7]. The SNP -Q551R of IL-4R (rs1801275) and susceptibility to periodontitis have also been discussed [7]. However, due to the limited sample size of individual studies and difference in population ethnicity, inconsistent findings were reported. In the present study, we systematically searched and evaluated case-control studies addressing the association between IL-4 and IL-4R polymorphisms and periodontitis susceptibility. Moreover, we performed meta-analyses in order to provide evidence with improved accuracy and less uncertainty.

## 2. Materials and Methods

The reporting of the present study follows the Meta-analysis Of Observational Studies in Epidemiology (MOOSE) statement [8].

### 2.1. Search Strategy and Study Selection

Databases including the PubMed, Embase, Scopus, ScienceDirect, Web of Science, CBM, CNKI, and WanFang were searched to identify relevant published papers addressing the association between IL-4 or IL-4R gene polymorphisms and periodontitis, up to August 1, 2016. The following search terms were used: “interleukin-4”, “interleukin 4”, “IL-4”, “Interleukin-4 receptor”, “IL-4R”, “periodontitis”, “periodontal disease”, and “polymorphism”. Full-text publications and their reference lists were carefully screened to decide whether information on the topic of interest was included. Additionally, the search was expanded by reviewing special meeting issues of journals in order to retrieve relevant abstracts. Studies that met the following criteria would be included: (i) case-control study on the IL-4 and IL-4R gene polymorphisms and susceptibility to periodontitis including chronic periodontitis (CP) and/or aggressive periodontitis (AgP); (ii) research subjects being patients with periodontitis and healthy controls; (iii) Reported data adequate for estimating the odds ratio (OR) with 95% confidence interval (95% CI); (iv) being published in English, Chinese, or Russian. On the other hand, studies with incomplete data and pedigree analysis and duplicated reports of the same study were excluded.

### 2.2. Data Extraction

Two investigators (Jia and Yuan) independently extracted the following data from each included study: first author's surname, publication year, country, ethnicity, type of disease, source of control, sample size, percentage of smokers among patients, genotyping method, genotype distribution in cases and controls, and Hardy-Weinberg equilibrium (HWE) for controls [9].

### 2.3. Quality Assessment

The included studies were evaluated in terms of methodological quality using the Newcastle-Ottawa scale (NOS) by two authors (Jia and Yuan) independently. Any discrepancy between the two authors was solved by discussion with a third investigator (Zeng).

### 2.4. Statistical Analyses

The Chi-squared test was used to assess the deviation of genotype distribution from HWE among controls. Crude ORs and corresponding 95% CIs were computed to assess the relationship between IL-4 and IL-4R polymorphisms and periodontitis susceptibility. The pooled ORs were calculated for the allele contrast, codominant, dominant, and recessive model, respectively. Heterogeneity was quantified using the *I*-squared statistic and assessed in terms of significance using the Chi-square based *Q*-test. *I*^2^ > 50% and/or *P* < 0.1 indicated significant heterogeneity among studies, in which case the random-effects model was used to perform the meta-analysis; otherwise, the fixed-effects model was used. Subgroup analyses were conducted with stratification by HWE, ethnicity, smoking status, and periodontitis type. Sensitivity analysis was performed to examine the robustness of the results. The potential publication bias was estimated by the modified Egger linear regression test. A 95% CI not crossing 1 was considered significant. All analyses were performed using the software R-3.3.1 (R Development Core Team, New Zealand).

## 3. Results

### 3.1. Study Selection and Characteristics

**Figure 1:**
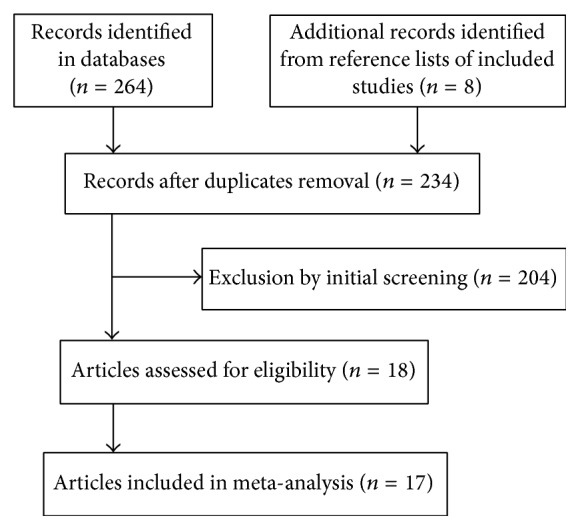
Flow chart showing the process from initial literature search to final inclusion of eligible studies.

As shown in Figure 1, we initially identified 264 articles. Finally, we included 17 articles [6, 7, 10–24] with 37 case-control studies involving 4,385 cases and 5,168 controls. Four polymorphisms of IL-4 gene and one polymorphism of IL-4R gene were included in our meta-analysis: fifteen on IL-4 -590C/T, eight on IL-4 -33C/T, two on IL-4 -1099T/G, eight on IL-4 70 bp VNTR, and four on IL-4R Q551R A/G. Three of these studies [17–19] contained data on two different subgroups (CP and AgP), two for a different ethnic population [15, 24], and one for a different smoking status among patients [10], which were considered independently in meta-analysis. In four articles with 5 case-control studies, the genotype distribution in control subjects did not conform to the HWE [11–13, 22]. Main characteristics of included studies were summarized in Table 1. All studies were of high quality (6-7 score). The main characteristics and summarization for quality assessment of included publications were shown in Table 1.

### 3.2. IL-4 -590C/T, -33C/T, and -1099T/G Polymorphisms and Periodontitis Susceptibility

Due to significant heterogeneity detected (Tables 2, 3, and 4), the random-effects model was used for all pooled analyses of the overall population.

**Figure 2:**
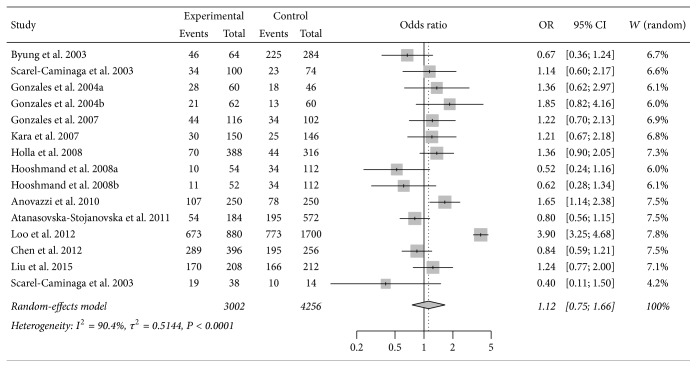
Forest plot for meta-analysis investigating the association between IL-4 -590C/T polymorphism and susceptibility to periodontitis, T versus C allele comparison in all study participants.

Meta-analysis of the IL-4 -590C/T showed no association between the polymorphism and periodontitis susceptibility (T versus C: OR = 1.12, 95% CI = 0.75–1.66; TT versus CC: OR = 1.44, 95% CI = 0.58–3.57; CT versus CC: OR = 1.26, 95% CI = 0.76–2.10; TT + CT versus CC: OR = 1.30, 95% CI = 0.74–2.26; TT versus CC + CT: OR = 1.2, 95% CI = 0.66–2.19). Subgroup analyses by disease type, ethnicity, and HWE status for controls were similar to the overall analyses (Table 2, Figure 2). According to the results of sensitivity analysis, the pooled result was not sensitive to any individual study except the one by Loo et al. [22]. Significant reduction in heterogeneity was observed after removal of this study, indicating that it was influential and might be an important source of overall heterogeneity. The details of sensitivity analysis were shown in supplementary table  1, in Supplementary Material available online at https://doi.org/10.1155/2017/8021279.

**Figure 3:**
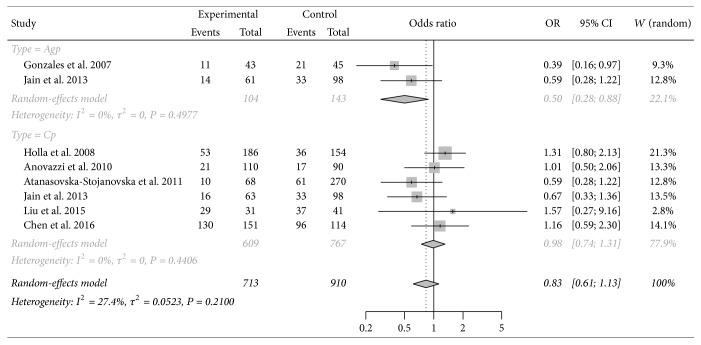
Forest plot for meta-analysis investigating the association between IL-4 -33C/T polymorphism and susceptibility to periodontitis. CT versus CC genotype comparison in subgroup analysis by periodontitis type.

Meta-analysis of the IL-4 -33C/T showed no association between the polymorphism and periodontitis susceptibility (T versus C: OR = 1.01, 95% CI = 0.69–1.47; TT versus CC: OR = 1.15, 95% CI = 0.57–2.34; CT versus CC: OR = 0.83, 95% CI = 0.61–1.13; TT + CT versus CC: OR = 0.92, 95% CI = 0.65–1.30; TT versus CC + CT: OR = 1.15, 95% CI = 0.57–2.34). Subgroup analyses according to disease type showed negative association with AgP (CT versus CC: OR = 0.50, 95% CI = 0.28–0.88) with no between-study heterogeneity (*I*^2^ = 0%) (Table 3, Figure 3). According to the results of sensitivity analysis, the pooled result was not sensitive to any individual study except the one by Holla et al. [17], and *I*^2^ decreased to 0% after this study removal, indicating that this study was influential and might be an important source of overall heterogeneity. The details of sensitivity analysis were shown in supplementary table 1.

Meta-analysis of the IL-4 -1099T/G showed no association between the polymorphism and periodontitis susceptibility (G versus T: OR = 1.2, 95% CI = 0.53–2.71; GG versus TT OR = 6.58, 95% CI = 0.03–11.46; TG versus TT: OR = 0.57, 95% CI = 0.32–1.04; GG + TG versus TT: OR = 0.73, 95% CI = 0.23–2.34; GG versus TT + TG: OR = 0.63, 95% CI = 0.03–11.35) (Table 4). Sensitivity analysis showed that the pooled result was not sensitive to any individual study. The details of sensitivity analysis were shown in supplementary table 1.

### 3.3. IL-4 70-bp VNTR Polymorphisms and Periodontitis Susceptibility

Due to significant heterogeneity detected (Table 5), the random-effects model was used for all pooled analyses of the overall population.

Meta-analysis of the IL-4 70-bp VNTR showed no association between the polymorphism and periodontitis susceptibility (2 versus 1: OR = 1.67, 95% CI = 0.71–3.96; 22 versus 11: OR = 1.39, 95% CI = 0.56–3.42; 12 versus 11: OR = 1.0, 95% CI = 0.43–2.31; 22 + 12 versus 11: OR = 1.09, 95% CI = 0.48–2.50; 22 versus 11 + 12: OR = 1.61, 95% CI = 0.89–2.93). The results of stratification analyses according to disease type and ethnicity were similar to the overall results (Table 5). Sensitivity analysis showed that the study by Anovazzi et al. [11] was influential, and *I*^2^ of the 12 versus 11 model decreased to 0% after its removal. The pooled result was insensitive to other individual studies. The details of sensitivity analysis were shown in supplementary table 1.

### 3.4. IL-4R Q551R Polymorphisms and Periodontitis Susceptibility

No significant heterogeneity was detected (Table 6); thus fixed-effects model was used for all pooled analysis of the overall population.

**Figure 4:**
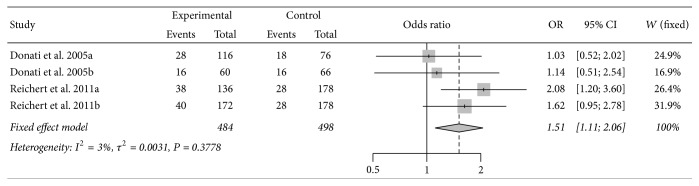
Forest plot for meta-analysis investigating the association between IL-4R Q551R polymorphism and susceptibility to periodontitis. T versus C allele comparison in all study participants.

Meta-analysis of the IL-4R Q551R polymorphism showed a positive association between the polymorphism and periodontitis susceptibility in three genetic models (R versus Q: OR = 1.59, 95% CI = 1.14–2.22; QR versus QQ: OR = 1.84, 95% CI = 1.21–2.80; RR + QR versus QQ: OR = 1.82, 95% CI = 1.22–2.72) with low between-study heterogeneity (Table 6, Figure 4). Subgroup analyses according to disease type and smoking status showed that the increased risk was predominant in CP and smokers (Table 6).

### 3.5. Publication Bias

Due to limitations of the quantity of included studies, we only tested the publication bias for IL-4 -590C/T polymorphisms. The funnel plots for T versus C allele model and TT versus (CC + CT) model suggested that there probably existed publication bias. Egger's test showed *P* values were *P* = 0.01 and *P* = 0.02, respectively, for T versus C allele model and TT versus (CC + CT) model. There is no publication bias in the other three genetic models.

## 4. Discussion

In the present study, we performed meta-analyses concerning the relationship between susceptibility and periodontitis (including CP and AgP) and polymorphisms including the IL-4 -590C/T, -33C/T, -1099T/G, 70-bp VNTR, and IL-4R Q551R, in the overall population and specific subgroups. Our study showed that the IL-4R Q551R R allele could significantly increase susceptibility to periodontitis in Caucasians, especially in terms of CP, which was more evident in those having a history of tobacco smoking. Similar findings were revealed with respect to the QR versus QQ and RR + QR versus QQ comparisons. The IL-4 -33C/T polymorphism was not associated with periodontitis susceptibility in the overall population; however, stratified analysis by type of disease showed that the CT genotypes were negatively associated with AgP. However, this association was nonsignificant as to CP. According to the results of overall meta-analyses and subgroup analyses by ethnicity, type of disease, HWE, and smoking status, no significant association was found between periodontitis susceptibility and polymorphisms IL-4 -590C/T, -1099T/G, and 70-bp VNTR, with the only exception that, in terms of the IL-4 -590C/T polymorphism, Caucasians with TT genotype showed marginally significant trend toward having periodontitis (TT versus CC: OR = 1.54, 95% CI = 0.99–2.41).

To the best of our knowledge, for the first time the IL-4 -1099T/G and IL-4R Q551R polymorphisms were included for meta-analytically analyzing the association between periodontitis susceptibility and gene polymorphisms, and the present study is the most comprehensive synthesis concerning polymorphisms on IL-4 and IL-4R and susceptibility to periodontitis. According to our results, it is demonstrated that variety exists in terms of IL-4 and IL-4R polymorphisms' distribution among different regions, populations, and disease types, and new evidence is produced. Compared to previously published meta-analysis, there are more studies included in the present meta-analysis, and the overall sample size is larger; therefore, our findings are more reliable. In addition, two more polymorphisms, that is, the IL-4-1099T/G and IL-4R Q551R, are discussed in our study, and it is revealed that the IL-4R Q551R allele may increase the susceptibility to periodontitis in Caucasians. Considering the complexity of the etiology and gene distribution of this disease, we performed comprehensive subgroup analysis stratified by ethnicity, disease type, and smoking status, by which our study topic was thoroughly analyzed.

Nevertheless, our study has some limitations. Firstly, although comprehensive literature search was conducted, certain gray literature could not be obtained, and therefore potential publication bias could not be excluded. Secondly, 5 studies inconsistent with HWE were included in our study, though sensitivity analysis found no significant influence on the overall results, and potential population bias could not be excluded. Significantly, major heterogeneity was found in most of the meta-analyses. Reduced heterogeneity was observed in some of the subgroup analyses. Given the complexity of periodontitis and potential confounding factors such as psychological stress and number of lost teeth, difference in clinical and/or environmental factors might have contributed to the heterogeneity among individual studies. Finally, the number of available studies was very limited in some subgroup analyses, and, due to the limited sample size, the pooled results were less accurate and more studies with large sample size and high quality are needed for further investigation.

To summarize, the present meta-analysis has demonstrated that a positive association exists between the IL-4R Q551R polymorphism and occurrence of CP. The IL-4 -33 CT genotype is negatively associated with the occurrence of AgP. However, this is not the case for other polymorphisms discussed. Our study findings provide guidance for early diagnosis and treatment of this disease. However, due to the limited number of studies included, additional studies with large sample size and adequate quality are needed to further verify and confirm our findings. Besides, interactions between multiple polymorphisms and between genetic and environmental factors should be investigated.

## Supplementary Material

In Supplementary Table 1 are shown collected resuts of sensitivity analyses. Individual influential studies are identified.

## Figures and Tables

**Table 1:** Characteristics of studies included in meta-analysis.

Study (author, year)	Country	Ethnicity	Disease type	Source	Sample Size case/control	Smoker %	Genotyping method	*P* for HWE	NOS score
IL-4 -590C/T									
Byung 2003 [7]	Korea	Asian	CP	HB	32/150	37.5	PCR	0.28	7
Scarel-Caminaga 2003a [24]	Brazil	Caucasian	CP	HB	50/37	0	PCR-RFLP	0.74	7
Scarel-Caminaga 2003b [24]	Brazil	Mixed	CP	HB	19/7	0	PCR-RFLP	0.43	7
Gonzales 2004a [15]	Europe	Caucasian	AgP	PB	30/33	NA	PCR-RFLP	0.18	7
Gonzales 2004b [15]	Japan	Asian	AgP	PB	30/31	NA	PCR-RFLP	0.09	7
Gonzales 2007 [6]	Germany	Caucasian	AgP	PB	58/51	12	PCR-RFLP	0.83	7
Kara 2007 [20]	Turkey	Caucasian	CP	PB	75/73	0	PCR-RFLP	0.48	7
Holla 2008 [17]	Czech	Caucasian	CP	PB	194/158	31.3	PCR-RFLP	0.53	7
Hooshmand 2008a [18]	Iran	Asian	CP	PB	26/56	0	PCR-RFLP	0.35	7
Hooshmand 2008b [18]	Iran	Asian	AgP	PB	27/56	0	PCR-RFLP	0.35	7
Anovazzi 2010 [11]	Brazil	Caucasian	CP	HB	125/125	15.2	PCR-RFLP	0.04	6
Atanasovska-Stojanovska 2011 [12]	Macedonia	Caucasian	CP	HB	92/286	0	PCR-RFLP	<0.05	6
Loo 2012 [22]	China	Asian	CP	PB	440/850	NA	PCR	<0.05	6
Chen 2012 [13]	China	Asian	CP	HB	198/128	NA	PCR	0.07	7
Liu 2015 [21]	China	Asian	CP	HB	104/106	27.9	PCR-RFLP	0.65	7
IL-4 -33C/T									
Gonzales 2007 [6]	Germany	Caucasian	AgP	PB	58/51	12	PCR-RFLP	0.67	7
Holla 2008 [17]	Czech	Caucasian	CP	PB	194/158	31.3	PCR-RFLP	0.53	7
Anovazzi 2010 [11]	Brazil	Caucasian	CP	HB	125/125	15.2	PCR-RFLP	<0.05	6
Atanasovska-Stojanovska 2011 [12]	Macedonia	Caucasian	CP	HB	92/286	0	PCR-RFLP	<0.05	6
Jain 2013a [19]	India	Dravidian	CP	PB	63/101	NA	PCR	0.63	7
Jain 2013b [19]	India	Dravidian	AgP	PB	61/101	NA	PCR	0.63	7
Liu 2015 [21]	China	Asian	CP	HB	104/106	27.9	PCR-RFLP	0.65	7
Chen 2016 [14]	China	Asian	CP	HB	440/324	0	PCR	1.12	7
IL-4 -1099T/G									
Chen 2012 [13]	China	Asian	CP	HB	278/324	NA	PCR	<0.05	6
Chen 2016 [14]	China	Asian	CP	HB	440/324	0	PCR	0.17	7
IL-4 70 bp repeat									
Byung 2003 [7]	Korea	Asian	CP	HB	32/150	37.5	PCR	0.27	7
Gonzales 2004a [15]	Europe	Caucasian	AgP	PB	30/33	NA	PCR-RFLP	0.70	7
Gonzales 2004b [15]	Japan	Asian	AgP	PB	30/31	NA	PCR-RFLP	0.16	7
Kara 2007 [20]	Turkey	Caucasian	CP	PB	75/73	0	PCR-RFLP	0.36	7
Holla 2008 [17]	Czech	Caucasian	CP	PB	194/158	31.3	PCR-RFLP	0.53	7
Anovazzi 2010 [11]	Brazil	Caucasian	CP	HB	125/125	15.2	PCR	0.80	7
Grigorovich 2015a [16]	Russian	Caucasian	CP	PB	150/150	NA	PCR	0.80	7
Grigorovich 2015b [16]	Russian	Caucasian	AgP	PB	150/150	NA	PCR	0.80	7
IL-4R Q551R									
Donati 2005a [10]	Swedish	Caucasian	CP	PB	60/39	50	PCR	0.44	7
Donati 2005b [10]	Swedish	Caucasian	CP	PB	30/34	0	PCR	0.32	7
Reichert 2011a [23]	Germany	Caucasian	CP	PB	68/89	25	PCR-SSP	0.52	7
Reichert 2011b [23]	Germany	Caucasian	AgP	PB	86/89	35.3	PCR-SSP	0.52	7

Mixed = Afro-Americans, Mulattos, and Asians. HB = hospital-based. PB = population-based. NA = not available.

Smoker % = smoker among patients %.

**Table 2:** Meta-analysis of the association between the IL-4 -590C/T polymorphism and periodontitis.

Genetic model and subgroup	Number of studies	Heterogeneity		Pooled results		
		*P*	*I* ^2^ (%)	OR	95% CI	*P* for OR
T versus C						
Overall	15	<0.01	90.4%	1.12	(0.75–1.66)	0.59
HWE (Y)	12	0.18	27.1%	1.02	(0.83–1.25)	0.84
Caucasian	9	0.07	45.3%	1.10	(0.86–1.39)	0.48
Non-Caucasian	6	<0.01	94.4%	1.20	(0.54–2.65)	0.66
CP	11	<0.01	92.7%	1.09	(0.68–1.77)	0.72
AgP	4	0.26	25.5%	1.17	(0.77–1.78)	0.45
TT versus CC						
Overall	16	<0.01	86.2%	1.44	(0.58–3.57)	0.44
HWE (Y)	12	0.26	19.0%	1.02	(0.62–1.66)	0.94
Caucasian	9	0.63	0.0%	1.54	(0.99–2.41)	0.06
Non-Caucasian	6	<0.01	94.1%	1.67	(0.21–13.57)	0.63
CP	11	<0.01	89.8%	1.41	(0.44–4.53)	0.57
AgP	4	0.42	0.0%	1.72	(0.78–3.80)	0.18
CT versus CC						
Overall	15	<0.01	81.0%	1.26	(0.76–2.10)	0.37
HWE (Y)	12	0.24	24.7%	1.00	(0.72–1.38)	0.98
Caucasian	9	<0.01	68.3%	1.02	(0.66–1.58)	0.91
Non-Caucasian	6	<0.01	85.6%	1.93	(0.51–7.27)	0.33
CP	11	<0.01	84.9%	1.90	(0.73–2.58)	0.39
AgP	4	0.16	42.3%	1.90	(0.48–1.89)	0.90
(TT + CT) versus CC						
Overall	15	<0.01	85.9%	1.3	(0.74–2.26)	0.36
HWE (Y)	12	0.21	23.7%	1.02	(0.75–1.38)	0.9
Caucasian	9	<0.01	63.9%	1.07	(0.72–1.58)	0.74
Non-Caucasian	6	<0.01	91.4%	1.83	(0.37–8.94)	0.45
CP	11	<0.01	89.3%	1.36	(0.67–2.78)	0.39
AgP	4	0.29	20.5%	1.06	(0.62–1.81)	0.84
TT versus (CC + CT)						
Overall	15	<0.01	85.8%	1.20	(0.66–2.19)	0.57
HWE (Y)	12	0.25	19.6%	1.02	(0.73–1.44)	0.90
Caucasian	9	0.46	0.0%	1.35	(0.88–2.06)	0.17
Non-Caucasian	6	<0.01	93.9%	1.11	(0.40–3.04)	0.84
CP	11	<0.01	89.5%	1.11	(0.55–2.27)	0.77
AgP	4	0.35	9.5%	1.83	(0.82–4.12)	0.14

Non-Caucasian = Asian and mixed.

**Table 3:** Meta-analysis of the association between the IL-4 -33C/T polymorphism and periodontitis.

Genetic model and subgroup	Number of studies	Heterogeneity		Pooled results		
		*P*	*I* ^2^ (%)	OR	95% CI	*P* for OR
T versus C						
Overall	8	<0.0001	83.2	1.01	(0.69–1.47)	0.98
HWE (yes)	6	0.0848	48.3	1.02	(0.78–1.33)	0.90
Caucasian	4	<0.0001	90.7	1.15	(0.57–2.34)	0.70
Non-Caucasian	4	0.0491	61.8	0.90	(0.61–1.32)	0.58
CP	6	<0.0001	83.2	1.07	(0.68–1.68)	0.76
AgP	2	0.0907	65.1	0.80	(0.39–1.67)	0.56
TT versus CC						
Overall	8	<0.0001	90.7	1.15	(0.57–2.34)	0.65
HWE (yes)	6	0.5117	0	1.32	(0.82–2.12)	0.26
Caucasian	4	<0.0001	90.3	1.57	(0.39–6.40)	0.53
Non-Caucasian	4	0.3466	9.3	1.06	(0.51–2.22)	0.87
CP	6	<0.0001	84.7	1.28	(0.45–3.62)	0.64
AgP	2	0.1579	49.9	0.94	(0.12–7.44)	0.95
CT versus CC						
Overall	8	0.2100	27.4	0.83	(0.61–1.13)	0.24
HWE (yes)	6	0.1373	40.2	0.84	(0.56–1.25)	0.39
Caucasian	4	0.0766	56.2	0.81	(0.48–1.36)	0.42
Non-Caucasian	4	0.4519	0	1.81	(0.54–1.20)	0.30
CP	6	0.4406	0	0.98	(0.74–1.31)	0.92
AgP	2	0.4977	0	0.50	(0.28–0.88)	0.02
(TT + CT) versus CC						
Overall	8	0.0301	54.8	0.92	(0.65–1.30)	0.62
HWE (yes)	6	0.1742	35	0.91	(0.63–1.30)	0.59
Caucasian	4	0.0186	70	1.00	(0.60–1.64)	0.98
Non-Caucasian	4	0.2462	27.6	0.80	(0.50–1.28)	0.35
CP	6	0.0352	58.2	1.03	(0.69–1.54)	0.88
AgP	2	0.5803	0%	0.62	(0.37–1.04)	0.07
TT versus (CC + CT)						
Overall	8	<0.0001	90.7	1.15	(0.57–2.34)	0.65
HWE (yes)	6	0.5117	0	1.32	(0.82–2.12)	0.26
Caucasian	4	<0.0001	90.3	1.57	(0.39–6.40)	0.53
Non-Caucasian	4	0.3466	9.3	1.06	(0.51–2.22)	0.40
CP	6	<0.0001	84.7	1.28	(0.45–3.62)	0.64
AgP	2	0.1579	49.9	0.94	(0.12–7.44)	0.95

*Note*. Non-Caucasian = Asian, Dravidian.

**Table 4:** Meta-analysis of the association between the IL-4 -1099T/G polymorphism and periodontitis.

Genetic model and subgroup	Number of studies	Heterogeneity		Pooled results		
		*P*	*I* ^2^ (%)	OR	95% CI	*P* for OR
G versus T						
Overall	2	0.48	0	1.2	(0.53–2.71)	0.66
GG versus TT						
Overall	2	<0.01	86.7	6.58	(0.03–11.46)	0.73
TG versus TT						
Overall	2	0.09	65.7	0.57	(0.32–1.04)	0.07
(GG + TG) versus TT						
Overall	2	<0.01	93.7	0.73	(0.23–2.34)	0.60
GG versus (TT+TG)						
Overall	2	<0.01	85.9	0.63	(0.03–11.35)	0.75

**Table 5:** Meta-analysis of the association between the IL-4 70 bp VNTR polymorphism and periodontitis.

Genetic model and subgroup	Number of studies	Heterogeneity		Pooled results		
		*P*	*I* ^2^ (%)	OR	95% CI	*P* for OR
2 versus 1						
Overall	8	<0.01	94.4	1.67	(0.71–3.96)	0.24
Caucasian	6	<0.01	95.8	1.90	(0.64–5.60)	0.25
Asian	2	0.0841	66.5	1.12	(0.46–2.70)	0.81
CP	5	<0.01	96.3	1.76	(0.53–5.85)	0.35
AgP	3	<0.01	88.6	1.54	(0.44–5.34)	0.50
22 versus 11						
Overall	8	0.01	61.7	1.39	(0.56–3.42)	0.48
Caucasian	6	0.01	66.9	1.66	(0.56–4.94)	0.36
Asian	2	0.1	56.0	0.79	(0.12–5.13)	0.80
CP	5	0.05	58.7	1.34	(0.53–3.43)	0.54
AgP	3	0.01	76.5	1.63	(0.11–23.69)	0.72
12 versus 11						
Overall	8	0.01	76.0	1.00	(0.43–2.31)	1.00
Caucasian	6	<0.01	79.6	0.97	(0.36–2.65)	0.95
Asian	2	0.07	69.2	1.09	(0.15–7.72)	0.94
CP	5	<0.01	82.2	0.82	(0.29–2.26)	0.69
AgP	3	0.16	45.2	1.68	(0.37–7.90)	0.51
(22 + 12) versus 11						
Overall	8	<0.01	78.1	1.09	(0.48–2.50)	0.84
Caucasian	6	<0.01	81.7	1.12	(0.41–3.06)	0.83
Asian	2	0.05	72.9	0.99	(0.13–7.29)	0.99
CP	5	<0.01	82.5	0.90	(0.34–2.38)	0.83
AgP	3	0.05	66.8	1.75	(0.24–12.67)	0.58
22 versus (11 + 12)						
Overall	8	0.0003	74.1	1.61	(0.89–2.93)	0.12
Caucasian	6	0.0313	59.2	1.65	(0.96–2.85)	0.07
Asian	2	0.55	0.0	0.86	(0.43–1.74)	0.68
CP	5	0.02	67.3	1.66	(0.89–3.08)	0.10
AgP	3	0.0008	86.0	1.37	(0.26–7.22)	0.72

1 for 184 bp, 2 for 254 bp.

**Table 6:** Meta-analysis of the association between the IL-4 Q551R polymorphism and periodontitis.

Genetic model and subgroup	Number of studies	Heterogeneity		Pooled results		
		*P*	*I* ^2^ (%)	OR	95% CI	*P* for OR
R versus Q						
Overall	4	0.38	3.0	1.51	(1.10–2.07)	0.01
CP	3	0.22	33.2	1.42	(0.89–2.27)	0.15
AgP	1	NA	NA	1.62	(0.95–2.78)	0.08
Smokers	3	0.28	20.6	1.59	(1.14–2.22)	<0.05
Nonsmokers	1	NA	NA	1.14	(0.51–2.54)	0.75
RR versus QQ						
Overall	4	0.44	0.0	1.46	(0.64–3.35)	0.37
CP	3	0.38	0.0	1.13	(0.41–3.11)	0.81
AgP	1	NA	NA	2.46	(0.59–10.32)	0.22
Smokers	3	0.32	11.6	1.14	(0.51–2.54)	0.27
Nonsmokers	1	NA	NA	0.83	(0.12–5.61)	0.85
QR versus QQ						
Overall	4	0.67	0.0	1.79	(1.21–2.65)	<0.05
CP	3	0.52	0.0	1.92	(1.19–3.11)	<0.05
AgP	1	NA	NA	1.57	(0.80–3.05)	0.19
Smokers	3	0.49	0	1.84	(1.21–2.80)	<0.05
Nonsmokers	1	NA	NA	1.50	(0.52–4.36)	0.46
( RR + QR) versus QQ						
Overall	4	0.53	0.0	1.75	(1.21–2.54)	<0.05
CP	3	0.34	7.4	1.79	(1.13–2.83)	0.01
AgP	1	NA	NA	1.67	(0.89–3.15)	0.11
Smokers	3	0.39	0	1.82	(1.22–2.72)	<0.05
Nonsmokers	1	NA	NA	1.34	(0.49–3.66)	0.56
RR versus (QQ + QR)						
Overall	4	0.48	0.0	1.20	(0.55–2.64)	0.65
CP	3	0.47	0.0	0.90	(0.34–2.37)	0.83
AgP	1	NA	NA	2.15	(0.52–8.89)	0.29
Smokers	3	0.35	5.4	1.35	(0.56–3.25)	0.53
Nonsmokers	1	NA	NA	0.71	(0.11–4.60)	0.72
