# Supplementary material for: Association between IL-4 and IL-4R Polymorphisms and Periodontitis: A Meta-Analysis
Source: Dis Markers. 2017 Mar 14;2017:8021279. doi: 10.1155/2017/8021279 (PMC5368398; doi:10.1155/2017/8021279)
Supplement: Supplementary file 1 — In Supplementary Table 1 are shown collected resuts of sensitivity analyses. Individual influential studies are identified. [file 8021279.f1.pdf]

Supplementary table 1: Sensitivity analysis with Random effects model

Genetic Model	Meta-analysis	tau^2	I^2%	OR	95%-CI	p-value
**IL-4-590C/T**						
TT vs. CC	Omitting Loo 2012 [22]	0.17	26.4	1.28	[0.83-1.97]	0.26
	Overall pooled analysis	2.40	85.2	1.52	[0.83-3.59]	0.34
TT vs. (CC+CT)	Omitting Loo 2012 [22]	0.09	25.7	1.14	[0.83-1.57]	0.42
	Overall pooled analysis	0.95	84.9	1.30	[0.83-2.31]	0.38
**IL-4-33C/T**						
CT vs. CC	Omitting Holla 2008 [17]	0.00	0	0.74	[0.55-0.99]	0.05
	Overall pooled analysis	0.05	27.4	0.83	[0.61-1.13]	0.24
**IL-4 70bp repeat**						
12 vs. 11	Omitting Anovazzi 2010 [11]	0.41	49.3	1.35	[0.65-2.77]	0.42
	Overall pooled analysis	0.92	76	1.00	[0.43-2.31]	1.00
**IL-4-1099T/G**						
G vs. T	Omitting Chen 2013 [13]	0.00	NA	0.40	[0.27-0.60]	<0.01
	Omitting Chen 2016 [14]	0.00	NA	1.58	[1.16-2.15]	<0.01
	Overall pooled analysis	0.91	96.4	0.80	[0.21-3.07]	0.74
GG vs. TT	Omitting Chen 2013 [13]	0.00	NA	0.11	[0.01-0.91]	0.04
	Omitting Chen 2016 [14]	0.00	NA	2.20	[1.28-3.78]	<0.01
	Overall pooled analysis	4.06	86.7	0.58	[0.04-11.46]	0.72
TG vs. TT	Omitting Chen 2013 [13]	0.00	NA	0.43	[0.28-0.67]	<0.01
	Overall pooled analysis	0.12	65.7	0.57	[0.32-1.04]	0.07
(GG+TG) vs. TT	Omitting Chen 2013 [13]	0.00	NA	0.40	[0.26-0.62]	<0.01
	Overall pooled analysis	0.66	93.7	0.73	[0.23-2.33]	0.60
GG vs. (TT+ TG)	Omitting Chen 2016 [14]	0.00	NA	2.26	[1.32-3.88]	<0.01
	Overall pooled analysis	3.81	85.9	0.63	[0.03-11.35]	0.75
